# 

**Published:** 1883-03

**Authors:** 


### NOTICE.

The subscription price of the JOURNAL OF HEALTH has been reduced to ONE DOLLAR a year. Those who have sent in their subscriptions at the old rate will receive the JOURNAL two years.

POST-MASTERS, CLERGYMEN, SCHOOL TEACHERS, READING ROOMS, PUBLIC INSTITUTIONS AND LITERARY SOCIETIES will receive the JOURNAL for 50 CENTS a year. Any POST-MASTER who sends us

TWO yearly subscriptions, at one dollar each, will be entitled to a third copy FREE. On all subscriptions over two he is entitled to 25 cents each.

We shall feel well recompensed if those to whom we send sample copies of the JOURNAL OF HEALTH will, after reading them, kindly pass them to others to read.

Send us EVEN dollars in MONEY and FRACTIONS of a dollar in ONE CENT postage stamps.

### PRICES.

| | |
|---|---|
| HALL'S JOURNAL OF HEALTH, ONE YEAR, - - - | $1.00 |
| DR. HALL'S HEALTH AT HOME, - - - - - | 4.00 |
| THE NEW NATIONAL STANDARD DICTIONARY, - - | 1.00 |

All will be sent, post-paid, on receipt of FIVE DOLLARS.

ANY SUBSCRIBER who will, at the end of the year, send us 75 cents and the 12 numbers of the JOURNAL, can have them handsomely bound and the volume returned by mail, postage paid.

TO ADVERTISERS.—We receive no advertisement but such as we deem unexceptionable. There is no publication that can show a better list of advertisers.

OUR RATES are very reasonable for a publication with so large a circulation. Address

"HALL'S JOURNAL OF HEALTH,"
NEW YORK.

